# Inhibition of negative content—a shared process in rumination and reappraisal

**DOI:** 10.3389/fpsyg.2014.00622

**Published:** 2014-06-17

**Authors:** Noga Cohen, Shimrit Daches, Nilly Mor, Avishai Henik

**Affiliations:** ^1^Department of Psychology and the Zlotowski Center for Neuroscience, Ben-Gurion University of the NegevBeer-Sheva, Israel; ^2^School of Education, Hebrew University of JerusalemJerusalem, Israel

**Keywords:** emotion regulation, reappraisal, rumination, inhibition, emotional stimuli

People vary in how they cope with negative events. Some people become immersed in repetitive ruminative thinking concerning the event, whereas others employ reappraisal and attempt to interpret the event in less negative ways. Interestingly, although both reappraisal and rumination involve active processing of negative situations rather than avoiding their affective value, these two strategies lead to opposite outcomes. Whereas rumination is maladaptive and is a risk factor for psychopathology, reappraisal is adaptive and has been linked to emotional well-being (for a meta-analysis see Aldao et al., 2010). In the current paper, we examine a shared process that may play a role in both rumination and reappraisal. We suggest that inhibition of irrelevant, negatively valenced information while pursuing a goal or performing a task underlies both rumination and reappraisal. We present correlational and causal findings linking impaired inhibition of negative content with reduced reappraisal and with increased tendency to ruminate. We postulate possible pathways for the links between inhibition of negative content and these two emotion regulation strategies.

## Defining reappraisal and rumination

Rumination and reappraisal are two strategies used to regulate emotion. When people reappraise, they transform the meaning of an emotional situation by changing the way they think about it (Gross, 1998). In contrast, when people ruminate, they think in a repetitive fashion about their current distress, the reasons for it and its consequences (Nolen-Hoeksema, 1991). For example, reappraisers would think of ways to improve the manner in which they study for an upcoming exam after recently failing one, whereas ruminators would think repeatedly why they did not perform better on the exam. Both strategies involve attending to the emotional value of the situation, but they differ in their outcomes. Reappraisal has been associated with reduced negative and increased positive emotions, improved interpersonal abilities, high self-esteem and other positive outcomes (Gross and John, 2003). In contrast, rumination prospectively predicts symptoms of anxiety and depression as well as other psychopathologies such as substance abuse and bulimic behavior (Kocovski et al., 2005; Nolen-Hoeksema et al., 2007, 2008). Although reappraisal and rumination have seldom been compared directly, such a comparison found that children who were instructed to reappraise a sad film had better memory of the film and were more effective in regulating sadness than those who were instructed to ruminate about it (Davis and Levine, 2013).

## Inhibition of negative content as a shared process in rumination and reappraisal

Despite limited knowledge about the relationship between reappraisal and rumination, examination of each of these strategies separately may point to a possible shared cognitive mechanism. Accumulating evidence suggests a link between cognitive control, a high-order cognitive operation designed to enable goal-directed behavior, and emotion regulation strategies (Ochsner and Gross, 2005). Several cognitive control processes have been proposed as underlying factors in both reappraisal and rumination (including inhibition, working memory updating, and set shifting; McRae et al., 2012; Whitmer and Gotlib, 2013). In this paper we focus on inhibition processes, because accumulating evidence suggests that inhibition may have a causal role in both rumination and reappraisal (Cohen et al., 2014; Daches and Mor, 2014; Salas et al., 2014).

Inhibition refers to the process of suppressing, resisting, and ignoring interference from task-irrelevant information (Friedman and Miyake, 2004). Recently, research has begun to uncover the associations between valance-specific inhibition and emotion regulation abilities (Joormann, 2010). In this paper we focus on inhibition processes designed to attenuate reactions to negatively valenced information that is irrelevant to current goals. Specifically, we propose that deficits in the ability to inhibit negative content may leave ruminators stuck in a cycle of negative thinking, whereas good ability to inhibit emotional content allows reappraisers to look at a negative situation from another perspective.

The ability to inhibit negative information has been examined using tasks that require participants to ignore emotional content, such as the negative affective priming (NAP) and the anti-saccade tasks. In the NAP task, participants are requested to respond to a target stimulus and to ignore an emotional distractor that appears simultaneously with the target stimulus. On a following trial, the emotional distractor might become the target (e.g., Joormann and Gotlib, 2010; for review see Joormann, 2010).The delay in reaction time to an emotional stimulus that was previously ignored, reflects activation of inhibitory processes (Wentura, 1999). In the anti-saccade task, participants are requested to inhibit their reflexive tendency to orient their attention toward an abrupt cue. Similar to the NAP task, when the cue is emotional, the task can serve to measure inhibition of emotional content (e.g., De Lissnyder et al., 2011).

Another way to examine inhibition of negative content is by using tasks that recruit inhibitory process (e.g., flanker: Eriksen and Eriksen, 1974; stop-signal: Logan and Cowan, 1984) prior to, and thus independently from, the presented emotional stimulus. Thus, in such tasks, in contrast to the NAP and the anti-saccade tasks, participants are not requested to ignore the emotional stimuli, but only to perform the inhibitory task, and the emotional stimuli remain irrelevant to the task. This design enables researchers to examine the effect of inhibition of non-valenced stimuli on the processing of irrelevant emotional stimuli. An example is the arrow-flanker task in which a right or left pointing target arrow appears with two distractor arrows on either side. Distractor arrows can be congruent with the target arrow (i.e., point in the same direction) or incongruent (i.e., point in the opposite direction). The existence of distracting flanker arrows, and particularly incongruent distractors, leads to engagement of inhibitory processes that reduce interference generated by the distractors (Aron et al., 2007). In several studies (e.g., Cohen et al., 2011, 2012), we demonstrated that presenting an incongruent flanker stimulus prior to the appearance of a negative picture eliminates the interference caused by the picture to performance on a subsequent simple cognitive task (see also Kalanthroff et al., 2013).

The tasks reviewed above were used to examine the association between trait tendencies to engage in rumination or reappraisal and inhibition of negative information. Research using the NAP found that people who report habitual reappraisal demonstrate increased ability to inhibit negative information (Joormann and Gotlib, 2010). Similarly, presenting an incongruent flanker stimulus prior to a negative picture resulted in attenuation of emotional interference only among high reappraisers (Cohen et al., 2012). These findings are in line with imaging research that showed that asking people to reappraise negative stimuli results in increased activation in frontal regions associated with cognitive control (Ochsner et al., 2002; Ochsner and Gross, 2007, for review see Ochsner and Gross, 2005), that might be related to inhibition. Similarly, it has been recently reported that impaired inhibition following a left fronto-parietal lesion was associated with a remarkable difficulty in spontaneous reappraisal generation (Salas et al., 2014). Thus, converging evidence suggests that reappraisal is associated with improved ability to inhibit negative content. The opposite pattern has emerged for rumination. Several studies using the NAP and the anti-saccade tasks have shown that ruminators have difficulty inhibiting emotional content (e.g., De Lissnyder et al., 2011), even when controlling for levels of depressive symptoms (Joormann, 2006; Zetsche and Joormann, 2011; but for discrepant findings see Goeleven et al., 2006). In line with these behavioral findings, investigators have identified rumination-related patterns of brain activation, such as a higher functional connectivity between the left striatum and the left inferior frontal gyrus (IFG), a region that has been associated with inhibition (Kühn et al., 2013).

## Reciprocal causal links between inhibition of negative content, rumination, and reappraisal

Although the ability to inhibit negative content has been associated with both rumination and reappraisal, the causal direction of the link between inhibition and these regulation strategies is unknown. It is possible that people's tendency to reappraise or to ruminate affects their ability to inhibit irrelevant emotional content. Alternatively, the ability to use inhibition processes when encountering negative information or thoughts may determine whether one will use reappraisal successfully or will become submersed in rumination. Experimental research that manipulates inhibition of negative stimuli or regulation strategy (i.e., rumination or reappraisal) can distinguish between these alternative causal directions.

Only a few studies have manipulated the use of specific emotion regulation strategies in order to examine their effect on inhibition of negative information. While there is some evidence that instructing people to ruminate impairs inhibitory abilities (Philippot and Brutoux, 2008), the effect of reappraisal on inhibitory abilities, is still unknown. Moreover, no studies to date have examined whether instructing people to ruminate or to reappraise modulates their ability to inhibit emotional content. Therefore, the effects of emotion regulation on inhibition of emotional content await further exploration. Our work has recently examined the alternative causal direction, demonstrating that training people to inhibit negative information can affect rumination. Specifically, utilizing the cognitive bias modification (CBM) paradigm (Hertel and Mathews, 2011), we trained participants to exercise inhibition over emotional stimuli, and examined the effects of this training on rumination and depressed mood. In one study, participants were trained to pair recruitment of inhibitory processes (using incongruent flanker targets) with negative pictures. Compared to participants in the control condition, those in the experimental condition exhibited reduced state rumination following training. Furthermore, whereas in the control condition higher levels of trait rumination were associated with increased sad mood following training, this association was eliminated among participants in the experimental condition (Cohen et al., 2014). In another training study, we randomly assigned participants to four sessions of training to inhibit or attend to negative stimuli or to a sham-training condition, using a novel procedure that was based on the NAP task. Compared with those who received the sham training, those who were trained to attend to negative stimuli exhibited a significant decrease in inhibition of irrelevant negative content, and those who were trained to inhibit negative stimuli showed a trend toward improved inhibition of irrelevant negative content. Furthermore, those who were trained to inhibit negative stimuli reported reduced trait rumination following the training (Daches and Mor, 2014).

Thus, far, there are no direct findings regarding the effect of inhibition modification on reappraisal. Watson and Purdon (2008) used an attention-training paradigm and examined its effect on the ability to use reappraisal in order to reduce the unpleasantness of intrusive thoughts in a sample of university students who reported high levels of obsessive-compulsive symptoms. No differences were found between the attention training and control groups in participants' ability to reappraise the unpleasantness of intrusive thoughts. Possibly, these null findings can be attributed to the fact that this study used attention training procedure rather than a procedure that directly targets inhibition of emotional content.

**Figure 1:**
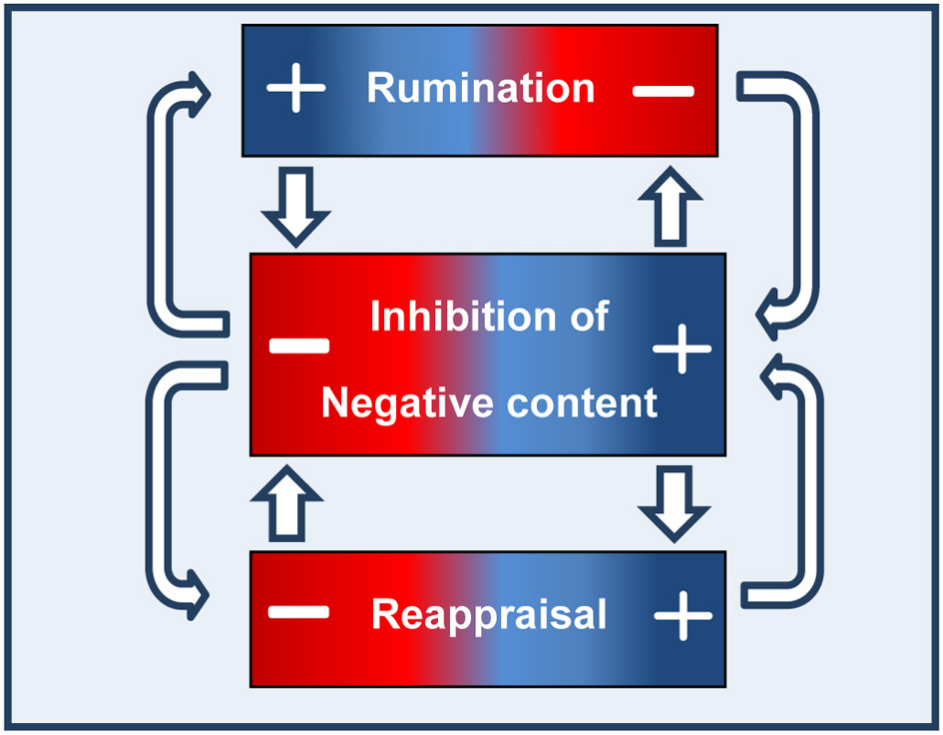
**The reciprocal causal links between the ability to inhibit negative content and rumination and reappraisal**.

To conclude, although research in this area is in its infancy, existing findings do suggest that the ability to inhibit negative content plays a casual role in emotion regulation, specifically in rumination and possibly in reappraisal. Limited evidence is available regarding the opposite causal direction—the influence of habitual rumination or reappraisal on individuals' ability to inhibit emotional content. Based on the available evidence, we propose a bidirectional relationship between the ability to inhibit negative content, and rumination and reappraisal, whereby inhibition plays a critical role in both rumination and reappraisal, and in turn, frequent use of these strategies may enhance or impair inhibition. As can be seen in Figure 1, elevated rumination level (as indicated by the + sign) impairs the ability to inhibit negative information, whereas elevated reappraisal level enhances it. Moreover, good ability to inhibit negative content prevents rumination and enables reappraisal. Indeed, recent findings of a study conducted by our group show that the ability to inhibit negative information is positively related to reappraisal only among low but not high ruminators (Daches and Mor, submitted).

Additional work is needed to fully understand whether rumination and reappraisal are on the same continuum or whether they constitute two distinct phenomena that share a common cognitive process. Resolving this debate will further our understanding regarding the cognitive operations that are at the basis of complex phenomena such as the use of different emotion regulation strategies. In addition, future studies should examine the causal link between other cognitive control processes, such as working memory updating and set shifting, and emotion regulation strategies (see for example Schweizer et al., 2013). Importantly, current and future studies focusing on strengthening inhibition processes or on training emotion regulation strategies, can lead to new interventions which would help individuals to successfully overcome stressful or unpleasant life events and avoid persistent psychological impairment.

### Conflict of interest statement

The authors declare that the research was conducted in the absence of any commercial or financial relationships that could be construed as a potential conflict of interest.
